# Co-creating opportunities to incorporate cessation for electronic nicotine delivery systems in family medicine – a qualitative program evaluation

**DOI:** 10.1186/s12875-021-01520-x

**Published:** 2021-08-24

**Authors:** Kevin A. Kovach, Reshana Peterson, Rajani Bharati, Kathryn Istas, Michael Monroe

**Affiliations:** American Academy of Family Physicians, 11400 Tomahawk Creek Parkway, Leawood, KS 66211 USA

**Keywords:** Family Medicine, Electronic Nicotine Delivery Systems, Electronic Cigarettes, Qualitative Research, Co-creation, Tobacco Cessation

## Abstract

**Background:**

The number of Americans who use tobacco has decreased in the twenty-first century, but electronic nicotine delivery systems (ENDS) have increased the complexity of treating tobacco dependence. The experiences of 18 family medicine practices were explored and opportunities to improve ENDS cessation were co-created in this study.

**Methods:**

Eighteen family medicine practices were enrolled into an implementation project to incorporate ENDS cessation into their practice. The participants’ experiences were explored throughout the project using an iterative qualitative approach. The research team provided technical assistance. Semi-structured group interviews and focus groups were held with participants at the beginning, middle, and end of the project to explore participants’ experiences. The collective knowledge and experiences of participants, expert consultants and the research team were fused together to co-create opportunities to improve ENDS cessation.

**Results:**

Nine opportunities to improve ENDS cessation were identified in three larger categories. The first category was leading change. This included: creating a vision for change to establish buy-in from key stakeholders and educate health care professionals to improve their confidence to address ENDS. The second category was creating processes. This included: establishing criteria for screening and quality improvement for ENDS cessation; being specific when asking about ENDS; creating electronic health record systems to support incorporating ENDS cessation; using chart audits if electronic health records cannot support incorporating ENDS into tobacco cessation; and assigning roles and responsibilities to members of the clinical care team. The third category was assisting patients who use ENDS. This included: educating patients and their parents/caregivers about ENDS and their potential harms, avoiding dual use, and developing a plan to quit.

**Conclusions:**

This study highlights challenges and opportunities for incorporating ENDS cessation into family medicine. The opportunities outlined here provide a practical approach which is rooted in the experiences of family physicians and their clinical care teams working to improve how they address ENDS and based on peer reviewed literature and expert input. Improving how ENDS are addressed in family medicine will require more than clinical expertise. It will also require leadership skills and the ability to create process improvements.

**Trial registration:**

Not applicable

**Supplementary Information:**

The online version contains supplementary material available at 10.1186/s12875-021-01520-x.

## Background

Great strides have been made to reduce the number of Americans who use tobacco in the twenty-first century. The percentage of adults who were regular smokers decreased from 20.9% in 2005 to 14.0% in 2019 [1], and there was also a reduction in middle school and high school students who reported being regular smokers, from 4.3 to 2.3% and 15.8 to 5.8% respectively, between 2011 and 2019 [2]. Despite this success, tobacco use remains the leading cause of preventable death, and more than 34.2 million people use cigarettes and other tobacco products in the United States [3].

Primary care plays a critical role in tobacco prevention efforts and could potentially contribute to half of the achievable reductions in cancer morbidity and mortality [4]. Approximately 20% of tobacco users who visit primary care are willing to attempt quitting [5], and there are effective behavioral and pharmacological treatments to help people quit [6, 7]. However, the tobacco use landscape has changed in recent years. Most notably, electronic cigarettes or electronic nicotine delivery systems (ENDS) have increased the complexity of treating tobacco dependence. One source of this complexity is that it is unknown if clinical recommendations for tobacco cessation work for ENDS cessation [8]. The patterns of ENDS use also differ among youth and adults. ENDS are the most used tobacco product among youth in the U.S. and in 2018 the U.S. Surgeon General declared e-cigarette use among youth an “epidemic” with ENDS largely replacing other forms of tobacco [9]. In contrast, ENDS use remains relatively low among adults and the majority of ENDS users are current or former smokers, suggesting that many adults use ENDS to quit or reduce tobacco use [10]. However, studies show that smokers who use ENDS often do not stop smoking cigarettes and are instead more likely to become dual users of both [11]. Dual users are at even greater risk of developing tobacco-related cardiopulmonary symptoms like chest pain and asthma [12]. To further complicate matters, there are varying estimates of benefits and harms due to ENDS at the population level. Some estimates suggest ENDS could reduce morbidity and mortality from tobacco use because of a potential net increase in smoking cessation. Other estimates suggest ENDS would increase morbidity and mortality from tobacco use due to the number of adolescent ENDS users who would potentially initiate cigarette smoking [13, 14]. While more research is needed, family physicians and their clinical care teams need better guidance on patient-centered approaches to address ENDS use among their patients now.

The American Academy of Family Physicians (AAFP) is the medical specialty society representing family physicians in the United States and its territories. The AAFP has a long history of working to reduce harms from tobacco use, including advocating for tobacco prevention policies and recommending that family physicians counsel all patients on the harms of tobacco use [15]. To support tobacco cessation in the primary care setting, the AAFP established the Ask and Act program, which is largely based on the U.S. Public Health Service clinical practice guideline, Treating Tobacco Use and Dependence: 2008 Update [15, 16]. In 2019, the AAFP launched the Reimagining Ask and Act for the 21^st^ Century program to identify challenges and opportunities to incorporating ENDS cessation among a sample of family medicine practices [17]. The questions addressed in this study included: (a) what challenges do family physicians and their clinical care teams experience incorporating ENDS cessation and (b) what opportunities are there to improve ENDS cessation? These questions are considered in the context of patient-centered care where cessation decisions should be made collaboratively between patients and their physicians and recovering from addiction is a challenging process.

## Methods

This study employed an iterative qualitative design. Aspects of implementation research and case study research were used to focus on how family physicians, their clinical care teams and other employees incorporated ENDS cessation in their clinical practices [18, 19]. An interpretivist approach was also used to recognize the different circumstances experienced by participants and to take into account different opinions about what constituted challenges and opportunities [20].

**Fig. 1:**
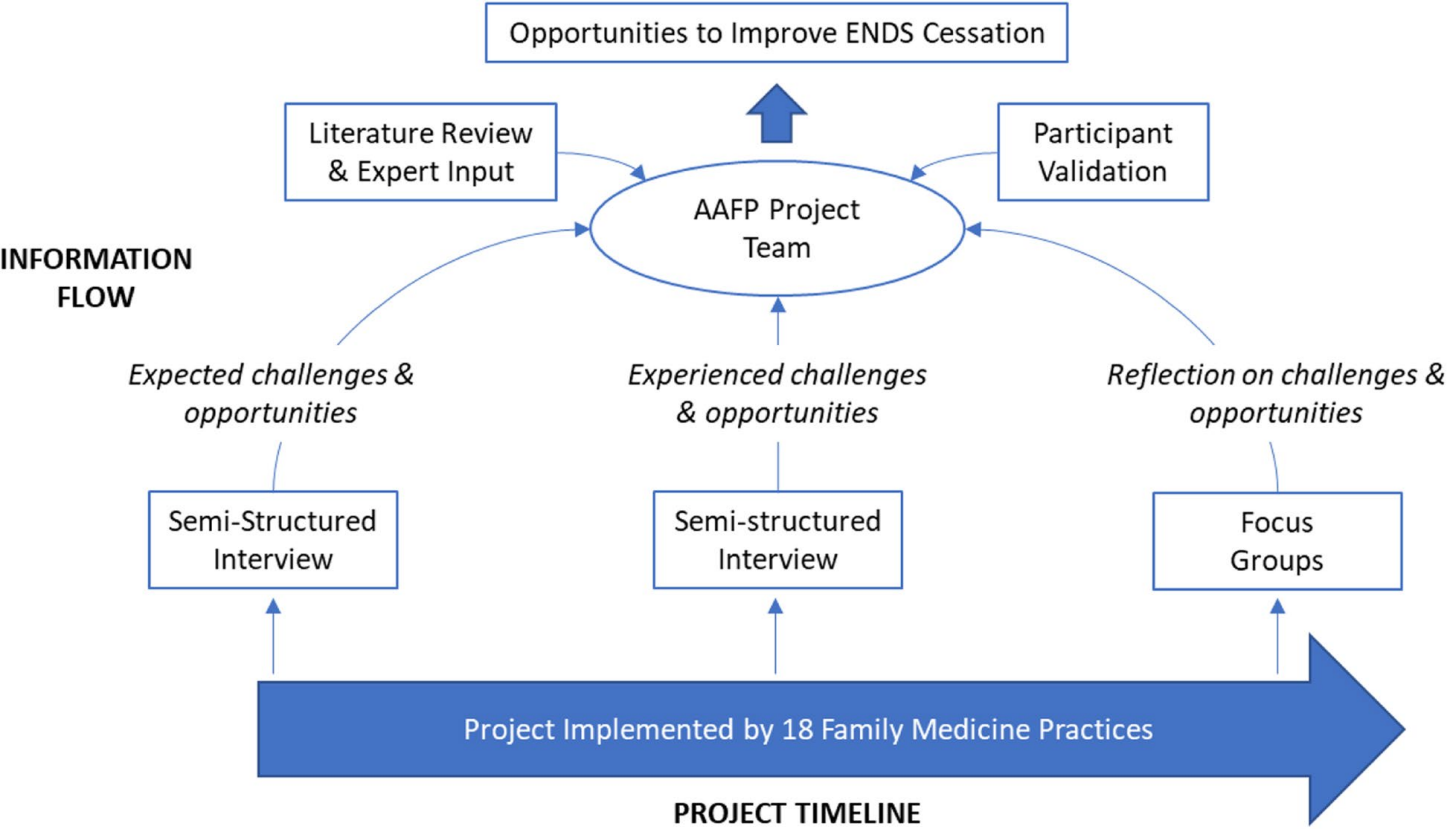
Research Design – Co-creating Promising Practices for Complete Nicotine Cessation

Overall, the experiences of family physicians, their clinical care teams and other employees from 18 family medicine practices were explored. Opportunities to improve ENDS cessation were defined as practices that would address participants’ challenges, could be operationalized coherently based on peer reviewed literature and expert opinion, and were viewed useful by participants. Data were collected primarily through semi-structured interviews and focus groups. Data were analyzed continuously throughout the study and findings from earlier phases informed future phases, which is in line with best practices for qualitative research [21]. The collective knowledge and experiences of participants, expert consultants and the research team were fused together to co-create opportunities to improve ENDS cessation (Fig. 1).

This study was reviewed by the AAFP’s Institutional Review Board and was expedited because it was a program evaluation limited to interview and survey methods. Written informed consent was obtained from all participants before the program began. Participants were reminded of their rights prior to qualitative data collection and verbal informed consent was obtained at this time. Risks to the participants were deemed low because the program focused on quality improvement for tobacco cessation. Participants’ confidentiality was assured by saving data on a password protected folder on AAFP’s network and the names of participants, their employers or other identifying information were not shared in any external communication. Datasets generated from the study are not publicly available to support confidentiality but could be obtained from the corresponding author on reasonable request.

### Reimagining ask and act for the twenty-first century: project design and implementation

#### Participant recruitment

The AAFP recruited family medicine practices for the Reimagining Ask and Act for the 21^st^ Century project in May and June 2019. Family medicine practices that were eligible employed a member of the AAFP, reported being able to use their electronic health records (EHRs) to document tobacco and ENDS use and cessation assistance, and treated both youth and adults. AAFP news articles, e-mails, newsletters, and social media were used to communicate the opportunity to join the project. Family medicine practices were provided a $5,000 stipend to offset administrative costs to implement the program. Each practice was asked to include at least one family physician and one non-physician employee to participate in the project.

#### Program implementation

Family medicine practices enrolled in the study were asked to identify a physician and non-physician champion to lead the project. The practices were provided with the AAFP’s Treating Tobacco Dependence Practice Manual, which includes resources to change clinical systems and culture to ensure that every patient who uses tobacco is identified, advised to quit, and offered evidence-based treatments [16, 22]. They were asked to extend this program to ensure that ENDS users were identified and provided cessation assistance as appropriate. The AAFP provided an hour-long orientation to the project participants using web conferencing software. The orientation addressed the current state of tobacco and ENDS use, risks to youth from using both tobacco and ENDS, current treatment recommendations for tobacco and nicotine dependence, and methods of screening, quality improvement, and leading organizational change. After the orientation, participants developed an implementation plan to include at least one clinical system change. Practices were then expected to incorporate the clinical systems change, test it, and refine their systems towards the goal of identifying and helping all tobacco and ENDS users. To support this, participants were to provide the research team monthly reports detailing the number of youth and adult patients who were served by the clinic, how many were asked about their tobacco and ENDS status, and the number provided assistance to quit if appropriate. This was intended to help the research team track both the practices’ progress and support their quality improvement process. Participants were given considerable autonomy regarding how they worked towards the program’s goals. The project began in June 2019 and ended in May 2020. The research team provided technical assistance throughout.

#### Data collection and analysis

Data were collected and analyzed in four phases. Eighteen group, semi-structured interviews were held with participants from each practice individually at the beginning and midpoint of the project. The purpose was to identify the challenges and opportunities participants expected and experienced, respectively. These interviews were conducted using web conferencing software and the data from the semi-structured interviews were used to identify challenges to incorporating ENDS cessation. This data informed the third phase of data collection, where six focus groups were held with family medicine practices that had previously discussed common themes identified by the research team. The focus groups were intended to identify additional detail and to begin to operationalize opportunities in a coherent manner for implementation. Opportunities for improvement were also elucidated through a literature review and consultation with a family physician expert on tobacco cessation. A draft of the findings was sent to participants to determine what opportunities they thought were useful and to identify additional insights to build on the opportunities, as a form of member checking in qualitative research [23].

The analysis was supported by Atlas.ti [24]. Data collection was followed by a process of transcribing, reading, and reflecting on the data. Two individuals from the research team (RP and RB) coded the transcripts individually and compared their analysis. This was shared with the full research team to seek understanding and consensus [25, 26]. For the purpose of this study, opportunities were defined as an “intervention, program/service, strategy, or policy that shows potential” to improve how ENDS cessation is incorporated into family medicine by addressing a challenge or using practices successfully implemented by the participants. We use this definition so as not to misconstrue these opportunities with best practices which require repeated demonstration of positive impact [27]. This is in alignment with Nesta’s level one standards of evidence, which states that “you can describe what you do and why it matters, logically, coherently, and convincingly” but not that “you have data that shows positive change [28].”

## Results

Eighteen family medicine practices participated in this study. This included four federally qualified health centers (FQHCs), two group practices, five health systems, and four university-owned practices. Seven of the practices served rural communities, four served suburban communities, and four served urban communities. The number of physicians working in each clinic ranged from 1 to 40 and the number of non-physician staff ranged from 6 to 89, with a median of 10 and 21 respectively. The number of patients ranged from 1,119 to 15,421, and the number of youth (ages 13–24) patients ranged from 104 to 2,735, with a median of 8,229 and 1,382 respectively. Family physicians were included from every practice. Non-physician participants included nurses, pharmacists, office managers, and quality improvement specialists based on how practices were organized. See Table 1 for information about the participating practices. Three practices did not report patient or youth patient counts.

**Table 1:** Participant characteristics of the reimagining ask and act for the twenty-first century program

Family medicine practices	Practice type	Urban/ Rural	Non-physicians	Physicians	Patients	Youth patients (Age 13 – 24)
Practice 1	FQHC	Rural	34	10	14,789	2126
Practice 2	FQHC	Suburban	8	15	1119	104
Practice 3	FQHC	Suburban	89	5	14,244	2735
Practice 4	FQHC	Urban	10	31	NR	NR
Practice 5	Group Practice	Suburban	32	5	3559	453
Practice 6	Group Practice	Rural	40	3	13,537	1502
Practice 7	Hospital/Health System	Rural	6	1	NR	NR
Practice 8	Hospital/Health System	Rural	11	2	5975	570
Practice 9	Hospital/Health System	Rural	9	1	NR	NR
Practice 10	Hospital/Health System	Suburban	15	5	6607	1,130
Practice 11	Hospital/Health System	Urban	32	26	8599	1482
Practice 12	Hospital/Health System	Rural	14	5	4020	416
Practice 13	Group Practice	Suburban	2	3	1647	318
Practice 14	Group Practice	Rural	16	1	3670	628
Practice 15	University-Owned	Rural	29	11	7858	1281
Practice 16	University-Owned	Urban	21	28	5312	128
Practice 17	University-Owned	Urban	20	40	12,020	2000
Practice 18	University-Owned	Rural	24	34	15,421	2438

*FQHC* Federally Qualified Health Center, *NR* Not Reported

In total, nine opportunities to improve ENDS cessation were identified within three larger themes: leading change; creating processes; and assisting patients who use ENDS. The overarching themes and specific opportunities for improvement are described in the following section. Overarching themes are described with an explanation of how each fit within the ENDS cessation process. Opportunities for improvement are described by providing the insights from the participants’ experiences from which they emerged and information about how to incorporate them into practice.

### Leading change

Leadership has been defined as selecting, equipping, training, and influencing followers to willingly and enthusiastically expend energy towards a goal [29]. Leadership is necessary to incorporate ENDS cessation into practice because ENDS are relatively new and assisting ENDS users to quit will require changes to clinical systems. It is imperative to equip primary care professionals with the leadership skills necessary to lead change [30]. Participants in this study uncovered both challenges and successes equipping, training, and influencing others to assist them with incorporating ENDS cessation into their practice. The most prominent leadership opportunities are described next.

#### Create a vision for change to establish buy-in from key stakeholders

Participants discussed the need to work with a variety of other stakeholders to incorporate ENDS cessation. This included health system leadership, EHR vendors, information technology (IT) teams, other physicians, and clinic staff. Making changes to EHR systems was one of the biggest challenges faced by participants. One participant said:

“Sometimes funding limits our ability to get access to certain [EHR] builds… just because it is not part of what the hospital would like… I think part of this project may be a need for increased advocacy in terms of getting [ENDS] embedded into more EHRs.”

The findings suggest that incorporating ENDS into an EHR is a multilayered problem and should be addressed at different levels. Currently, ENDS has not been included in many EHR systems and recommendations have been made to expand this [31]. This could be addressed directly by advocating for EHR vendors to include ENDS in routine builds. It also suggests that hospitals and health systems could request ENDS to be included in their EHR. But this may incur a cost and may require allocation of IT personnel, creating the need for buy-in from leadership and IT. Either way, a compelling vision is needed to create change.

Participants also discussed issues regarding the engagement of other health care professionals in their practice and broader health care system. Most participants noted the importance of establishing buy-in from internal staff, as they are crucial to making changes. One participant said, “The staff buy-in is really important… They’ve got so many things they have to do… and we’re adding yet another burden… we have to make sure it’s portrayed as important.” Participants appeared to mostly do a good job of engaging other staff members. However, participants also discussed opportunities to improve the reach of ENDS cessation to other physicians and clinicians outside of their immediate practice. One participant said, “I’m just one part of a very large multi-specialty group practice. I have a lot of people that we could roll this out to.” However, most participants said they had not worked to influence how ENDS were addressed with other physicians or other practice settings, suggesting there may be gaps spreading promising practices throughout health care systems.

Creating a vision for why incorporating ENDS cessation is important and may help to establish buy-in from key stakeholders. A vision for change is a description of a better future state toward which stakeholders would willingly and enthusiastically work [29]. Kotter suggests that a vision should be ambitious, reduce complacency, appeal to customers (or patients), and take advantage of recent trends [32]. While most participants could articulate goals for their project, they tended to focus on processes, like “routinization,” “data-driven decision making,” and “engaging the entire health care team.” It is unclear if these types of goals would motivate key stakeholders to assist with making the changes to incorporate ENDS cessation into practice because they are not necessarily ambitious, patient-focused, and do not take advantage of recent trends around ENDS use like vaping-induced lung injury or COVID-19 risk [33, 34]. A compelling vision for ENDS cessation may include local data about ENDS use in the community, local stories even about a single patient, or personal anecdotes from health care professionals. For example, one of the participants discussed finding their child’s vaping device and how that raised their awareness and concerns about ENDS in the community.

#### Educate health care professionals to improve their confidence to address ENDS

Participants expressed a lack of knowledge about ENDS among themselves and their colleagues and suggested this was a barrier to successfully incorporating ENDS cessation into practice. One participant arranged training for the staff in her clinic and commented on how few were knowledgeable about ENDS, saying, “There were maybe two people in a room of fifteen plus medical professionals who knew anything about [ENDS]… I think my colleagues who aren’t asking… are totally in the dark.” Another participant noted that there was not readily available education about ENDS for healthcare professionals, saying, “There isn’t a set curriculum… and I think [with a set curriculum] we would all know what everyone is doing and that we’re all trying to do the same thing.” These kinds of comments were relatively common among participants and suggest that all employees with a role in ENDS cessation require education about ENDS.

Creating a standardized curriculum in family medicine practices, residency training, and continuing medical education to increase knowledge of ENDS could improve the confidence and routinization of primary care professionals to address ENDS use with patients. Topics that need to be covered include the health risks of ENDS, current terminology or “lingo,” the screening and treatment process, motivational interviewing, and quality improvement. Participants suggested that this education needed to be offered frequently and “streamlined… and more standard” to ensure that all employees had the same education and were expected to be able to play their role.

### Creating processes

Consistent screening and quality improvement processes are needed to incorporate ENDS cessation into practice. Screening refers to systematically identifying and documenting individuals who use ENDS, and providing them appropriate assistance [35]. Similarly, quality improvement refers to a systematic and cyclical process of setting goals, measuring progress, and identifying and testing interventions to meet the established goals [36]. Clinical recommendations, policies from medical specialty societies, and statements from the Surgeon General support screening for tobacco and ENDS [6, 7, 9, 37]. However, there are inconsistencies which may make these processes challenging to implement. The participants in the Reimagining Ask and Act for the 21^st^ Century Program experienced several challenges while screening for and performing quality improvement to incorporate ENDS cessation. These opportunities are described next.

#### Establish criteria for screening and quality improvement for ENDS cessation

Screening and quality improvement for ENDS use requires assigning specific criteria to monitor the proportion of patients who are asked about their use and provided appropriate assistance. While the participants in this study did establish age ranges for screening and quality improvement, it did not appear that they all established standardized treatment options for different population groups. This may be understandable as there are different treatment recommendations for tobacco use among youth, adults, and pregnant individuals and ENDS users from the USPSTF [6, 7]. But it may also be problematic because it will not be possible to conduct quality improvement processes on tobacco and ENDS cessation treatment if criteria for the preferred course of action is not made explicit.

There was substantial variation in the age range used for tobacco and ENDS screening. Before the project started, some participants began screening at age 11 and others did not start screening until age 18. Some participants discussed that it was unclear at what age to begin screening for tobacco and ENDS use. One participant captured this saying:

“I don’t get the impression that [health care professionals] understand how common [ENDS use] is among… middle school students. I think if [health care professionals] are making assumptions, they’re thinking high school and they’re surprised at the middle school age kids doing this.”

Clinical recommendations do not provide much additional clarity, stating that interventions should begin with “school age children and adolescents,” but do not provide a specific age range [7, 17]. However, data from the National Youth Tobacco Survey suggests that screening for tobacco and ENDS use should, at minimum, begin by age 12, since 28 and 7% of 16–17 year old tobacco and ENDS users began using at or before age 12, respectively [38]. This is a substantial number, when also considering that earlier age of initiation is associated with stronger nicotine dependence throughout life [39].

Criteria for screening and quality improvement for ENDS cessation should identify patient characteristics and standards of care. We suggest the following criteria shown in Table 2 based on clinical recommendations from the USPSTF, AAFP policy, the Surgeon General, and other published literature [6, 7, 9, 37].

**Table 2:** Screening and quality improvement criteria for tobacco and ENDS cessation

Patient characteristics	Standards of care
Youth: ≤ 12 to 17 years old	- Provide a confidential space for youth by asking parents/guardians to leave the room. Disclose positive screens only after getting permission from patients
	- Ask and document tobacco and ENDS use status
	- If no, provide education to prevent tobacco and ENDS use
	- If yes, use clinical judgement to determine how best to assist:
	○ Behavioral counseling: The USPSTF found insufficient evidence to support behavioral counseling for tobacco and ENDS in youth, but the harms of behavioral counseling are likely to be small [40]
	○ Pharmacotherapy: The USPSTF found no evidence supporting the use of medications to improve tobacco or ENDS cessation among youth [40]
Adults: 18 years old or older	- Ask and document tobacco and ENDS use status
	- If yes, advise them to stop, provide behavioral interventions and FDA approved pharmacotherapy [6]
Pregnant: 18 years old or older	- Ask and document tobacco and ENDS use status
	- If yes, advise them to stop and provide behavioral interventions [6]

#### Be specific when asking about ENDS

There was also substantial variation in the terminology participants used to ask patients about ENDS use. While participants understood the importance of using terms familiar to patients, many were not current, or could not stay current, with the quickly evolving language. Some participants thought this was a barrier to incorporating ENDS cessation, and one participant said: “I think [health care professionals] all say their own thing. I’m not sure they’ve ever been educated exactly how to ask [about ENDS].” Research underscores the importance of using a broader set of terms to identify ENDS use. Asking only about e-cigarettes misses 33% of ENDS users, as many youth do not associate vaping with the use of e-cigarettes [41]. Misclassification was greatest among females, minorities, and individuals who had not used traditional tobacco products, which could create health disparities [41]. The Truth Initiative created the Vaping Lingo Dictionary in 2020 which can assist with this challenge. This includes the names of most popular brands of ENDS, as well as up-to-date terminology and slang [42].

Based on this information, health care professionals should ask about multiple types of ENDS products to maximize the number of ENDS users identified. The Truth Initiative’s Vaping Lingo Dictionary or similar resources should be used to identify terms [42]. Since language varies by region, surveys or interviews could be conducted by local health departments or community-based organizations to identify local slang/terminology. Organizations that work with youth, such as schools or public health departments may also be able to provide insight into local slang.

#### Create EHR systems to support incorporating ENDS cessation

Most participants experienced considerable challenges modifying their EHRs to support ENDS cessation. Typically, IT personnel were needed to manually modify EHRs, which could take up to several months. Participants discussed how EHRs were not being maximized, saying, “The functionality of the EHR, it’s being so underutilized in terms of what it’s capable of.” But others explained how setting up an EHR system appropriately was challenging, saying,

“Older EHRs don’t have the ability to make that integration or add questions… they are building whole new screens… whole new parts of their system. So it is more complicated. And there are some [EHRs] that don’t even have the ability to make those changes.”

Participants who were able to modify their EHR reported their ENDS screening and assistance rates more consistently to the research team, suggesting they were more capable of studying the effects of their implementation plan for quality improvement. These participants also viewed EHRs more positively, saying: “I think one of the most helpful things to keep consistency of the ask and the documentation was when we incorporated it into the electronic medical record.” Suggestions include:

- Health informatics companies should ensure that tobacco and ENDS are incorporated into their EHR systems in a way that integrates both as forms of tobacco, and that allows for easy documentation and reporting.
- Buy-in and support from IT personnel should be sought early in the absence of appropriate EHR set up. The vision for incorporating ENDS into tobacco cessation, as described earlier, may help IT personnel to understand why this important. A clear description of how to set up the EHR may help to ease the work needed by IT staff. Both could help support buy-in.
- EHRs should be set up to support screening, documentation, assistance, and reporting.
  - Prompts should be used to facilitate screening and assistance. Research shows that prompts improve quality of care and some of the participants successfully used prompts to incorporate ENDS into tobacco cessation [43].
  - Structured response fields (checkmarks, yes/no) should be used instead of unstructured fields (text entry) whenever possible to standardize screening, documentation, and allow for automated reporting.
  - Multiple specific fields should be used to document the natural history for quitting tobacco and ENDS. Research shows that documentation in this manner is associated with more consistent screening and cessation assistance [44]. Some participants discussed successes adding structured response fields, saying: “we added a drop-down box within the vaping use section so that we can specify the device type, the frequency of use, … patient strengths, … reasons for vaping, …and past attempts at cessation.” Numerous fields for ENDs have been identified for use in documentation, including for: use (ENDS use, type of product used, frequency and amount used), treatment (advised to quit, counseling provided, referral to treatment or Quitline, medication prescribed, and patient education provided), and patient outcomes (willingness to quit, number of quit attempts, changes in ENDS use status) [44–46].

### Use chart audits if EHRs cannot support incorporating ENDS into tobacco cessation

As stated previously, most participants experienced considerable challenges modifying their EHRs. Some were never able to make the appropriate changes, which inhibited their efforts in conducting quality improvement. One participant captured this saying: “I have not been able to get that [data] output from our EHR… I’m a little bit reluctant to even go with an intervention until I have that data.” Chart audits could be used to overcome challenges with EHRs [47]. Focus needs to be placed on easing data collection and analysis, while maintaining high standards for data quality. This will ensure that the chart audit process is not too burdensome to complete regularly, and that the data are still trustworthy to inform changes to the practice system. Suggestions include:

- Rapid cycle quality improvement cycles should take place at least every three months to support gradual but persistent improvement [48]. Suggested steps for using chart audits for quality improvement include: specifying the goal; identifying inclusion and exclusion criteria; defining the time period for review; stratifying by factors that may impact the trustworthiness of the results (clinic site for example), determine the sample size, and collect, organize, and analyze the data [49, 50].
- Use a sample rather than all patients. One participant manually reviewed every patient record for this project, but this type of review is too cumbersome to support long-term success and success at scale. Differences between performance and goals can typically be identified with a sample of 25 patients (range 5 to 280) with enough power for statistical significance [50]. A larger sample will be needed when differences between performance and goals are small.

#### Assign roles and responsibilities

Participants discussed the importance of assigning roles and responsibilities for ENDS cessation throughout the entire health care team. One participant said: “It really has more to do with embedding [ENDS cessation] within the workflow… I think… that would be the most potent process improvement we could make.” Another participant discussed the types of roles and responsibilities that were assigned, saying: “Our clinical assistants… are actually involved in either taking vitals and bringing patients back… We have added asking about tobacco and vape use at every visit as part of our vital signs.” Roles and responsibilities for screening, patient care, and quality improvement all need to be assigned so that team members understand how they contribute to ENDS cessation.

### Helping patients quit ENDS

Ultimately, the purpose of incorporating ENDS cessation is to prevent use and help patients who use ENDS to quit. However, the evidence is not clear how to help individuals who use ENDS to quit and clinical judgement is often required to determine how best to help. One participant captured how complex helping individuals that use ENDS is, stating: “If a patient says I’m interested in quitting… that takes the guesswork out of it. But I don’t know what the process is for a patient who says they are smoking or vaping [but are not ready to quit].” Since evidence is still inconclusive about how well interventions for tobacco work for ENDS, the identified opportunities we present next provide direction for helping patients who use ENDS and encourage substantial flexibility for clinical decision making.

#### Educate patients and their parents/caregivers about ENDS and potential harms

Many patients, along with their parents and family members, have misconceptions about ENDS and are unaware of their potential harms. One participant shared, “I think e-cigarettes are assumed by many [to be safe].” In general, the aerosols produced by e-cigarettes contain far fewer chemicals than are found in smoke from combustible tobacco products [51]. Because of this, e-cigarettes may be considered less harmful than combustible cigarettes, but that does not mean that they are safe [52, 53].

E-cigarettes contain nicotine, which is an addictive chemical derived from tobacco. Newer cartridge-type e-cigarettes can contain as much nicotine as an entire pack of regular cigarettes [54]. The dramatic increase in e-cigarette use among U.S. youth in 2018 was immediately preceded by the introduction of flavored forms of these high-nicotine-content products to the e-cigarette product market [55, 56]. The role of nicotine addiction in sustained use of tobacco is well-documented, as is the increased susceptibility of adolescent brains to harms caused by early nicotine exposure – including greater risk of long-term nicotine addiction [51, 57]. Some studies comparing dependence associated with use of e-cigarettes and combustible cigarettes have found e-cigarettes to be consistently associated with lower nicotine dependence than cigarette smoking [58]. However, these studies focused only on adult e-cigarette users and relied on data collected before cartridge-type, high-nicotine-content products entered – and quickly dominated – the e-cigarette market [55]. Nicotine is also toxic to developing fetuses and can seriously harm adolescent brain development [51].

ENDS vapor contains numerous cancer-causing chemicals and potentially harmful microparticles that are inhaled deep into the lungs [51]. The variability in products makes identifying the number, quantity, and characteristics of these potentially harmful substances difficult [59]. Many of the chemical flavorants and solvents used in e-cigarette liquids are not readily disclosed [51, 60, 61]. Some potential effects of ENDS use include vomiting, mouth and airway irritation, chest pain, and palpitations; increased heart rate, blood pressure and arrhythmias; exposure to carcinogens; acute lung injury; seizures; and increased risk of more serious upper respiratory conditions such as COVID-19 [33, 34, 62, 63]. Although many of these effects may be temporary and dissipate with continued use, there is very little known about the long-term health effects of e-cigarette use – particularly among youth [59].

Patients should be educated to correct these and other misconceptions about the safety of e-cigarettes. While ENDS are not approved by the Food and Drug Administration for tobacco cessation [15], some patients continue to use ENDS as a cessation aid. Some research suggests that even though some people may use ENDS to quit using tobacco, many people become dual users or addicted to ENDS [62]. Patients may also be unaware that dual use of ENDS and tobacco may actually be riskier than tobacco use alone [64].

### Avoid dual use and develop a plan to quit

ENDS are not FDA approved for tobacco cessation and should not be recommended as a cessation device. However, many individuals use ENDS as a cessation device and family physicians and their clinical care teams will be faced with providing them care [65]. Understanding how to help patients plan to quit using tobacco and ENDS is important. There are many different patterns of ENDS use and some may be less aligned with a planned approach to complete tobacco and ENDS cessation than others [66]. Patterns of ENDS use that may be more prone to tobacco and ENDS cessation are characterized by switching entirely from tobacco to ENDS and having a plan to quit [66]. This may include quitting tobacco and using ENDS occasionally when cravings are high, gradually weaning off ENDS by lowering nicotine dosages, or gradually transitioning from tobacco to ENDS, then to FDA approved medication for quitting. Patterns of ENDS use that may be less prone to cessation are characterized by dual use of tobacco and ENDS without a plan to quit [66]. This might include using ENDS in places where smoking is not allowed; continuing to smoke in stressful situations; or using ENDS for recreation or enjoyment. For dual users of tobacco and ENDS, it may be worthwhile to transition them from dual use to using ENDS only, and then using FDA approved pharmacotherapy to complete cessation. Steps may include:

- Identify the patient’s pattern of ENDS use.
- Establish a plan to quit tobacco and ENDS. Include a quit date.
- Educate individuals that use tobacco and ENDS that dual use is at least as harmful as conventional tobacco smoking and may be worse. Transition dual users off tobacco products.
- Transition ENDS users to FDA approved cessation aids as appropriate.
- Transition to full cessation over time.
- Provide services like counselling, classes, behavioral health, and quitlines to support cessation.
- Develop a system to follow-up with ENDS users to evaluate cessation progress.

## Discussion

This study highlights challenges and opportunities for incorporating ENDS cessation into family medicine. The opportunities outlined here provide a practical approach which is rooted in the experiences of family physicians and their clinical care teams, that were working to improve how they address ENDS. The opportunities are also based on peer reviewed literature and expert input. Developing opportunities to improve implementation of ENDS cessation in this manner may be important, because ENDS use continues to escalate at a rate that is outpacing traditional epidemiological and health services research.

The aim of this research was to identify promising practices directly from the participants in this study; however, most of the identified opportunities emerged from their challenges. This resulted in a change in methodological strategy from one where solutions could be directly extracted from the participants to one where opportunities could be co-created from their experiences, the literature, and expert opinion. Ultimately, this was a positive change and reflects a strategy for the research team to use an action research approach where the roles of technical assistance and research is linked [67].

The themes emerging from this study focus on leadership, processes, and patient care. Improving how ENDS are addressed in family medicine will require more than clinical expertise. An understanding of how to lead through influence will be necessary to help establish buy-in from the various key stakeholders. Adaptive and visionary leadership strategies may be useful to help create a sense of urgency for change [30, 68]. Sufficient technical knowledge of EHRs can facilitate collaboration with EHR vendors and IT personnel vital to creating systems to support tobacco and ENDS cessation. In addition, an educational strategy that evolves with changes to ENDS products and research will be needed to stay up to date with the best practices.

The implications for policy and practice are varied. Most of these identified opportunities are not new, but as shown here, are underutilized. The AAFP will be using these findings to create a guide for ENDS cessation in 2021. This guide and others like it could help improve the diffusion of existing best practices for a patient-centered approach to cessation. Other identified opportunities shared here may be a bit controversial, as they are not supported by robust, systematic reviews or approved by the USPSTF or other governing bodies. However, research has not been able to keep up with the ENDS epidemic. In these circumstances, clinicians should use the best available evidence and patient desires to assist with clinical judgement, which can include the experience of their peers, such as presented here [69].

### Strengths and limitations

There are both strengths and limitations to this study. First, the findings are based on the experiences of a broad range of 18 family medicine practices, including physicians, nurses, quality improvement managers, and other health care professionals. Incorporating perspectives from a variety of participants helped us reach data saturation. Second, the data was limited to interviews and focus groups conducted with web conferencing software. Face-to-face meetings may have helped establish rapport more easily. However, the findings should be credible as we were able to create robust descriptions of the participants’ experiences and were able to triangulate between different family medicine practices and different types of participants. We also conducted member checks by asking participants to respond to a draft of this findings report. One of the biggest limitations was that this study took place during the COVID-19 pandemic. This created challenges for some practices to incorporate ENDS cessation as patient load decreased, or priorities changed. However, this did not affect every practice and most participants had already completed most of their project by this point.

## Conclusion

Family medicine professionals need strategies to incorporate ENDS cessation into daily practice. This study provides insight into opportunities to address ENDS use based on experience, expert opinion, and the best available evidence. The opportunities described here may help to reduce the complexity of addressing ENDS use in primary care, helping to improve treatment implementation. The findings provide a potential path forward to addressing ENDS use today. Future research is needed to better establish evidence-based practices to help patients quit using ENDS.

## Supplementary Information

**Additional file 1.** Semi-structured interview guide

## Data Availability

Datasets generated from the study are not publicly available to support confidentiality but could be obtained from the corresponding author on reasonable request.
