# Supplementary material for: Co-creating opportunities to incorporate cessation for electronic nicotine delivery systems in family medicine – a qualitative program evaluation
Source: BMC Fam Pract. 2021 Aug 24;22:169. doi: 10.1186/s12875-021-01520-x (PMC8382936; doi:10.1186/s12875-021-01520-x)
Supplement: Supplementary file 1 — Additional file 1. Semi-structured interview guide [file 12875_2021_1520_MOESM1_ESM.docx]

**Additional File 1.**

**Semi-structured interview guide**

**Program Title:** Reimagining Ask and Act for the 21^st^ Century Program

**Manuscript Title:** Co-creating Opportunities to Incorporate Cessation for Electronic Nicotine Delivery Systems in Family Medicine – A Qualitative Program Evaluation

**Authors**

Kevin A. Kovach, DrPH, MSc

American Academy of Family Physicians

11400 Tomahawk Creek Parkway

Leawood, KS 66211

[kkovach@aafp.org](mailto:kkovach@aafp.org)

Reshana Peterson, MPH

American Academy of Family Physicians

11400 Tomahawk Creek Parkway

Leawood, KS 66211

[rpeterson@aafp.org](mailto:rpeterson@aafp.org)

Rajani Bharati, MPH, RN

American Academy of Family Physicians

11400 Tomahawk Creek Parkway

Leawood, KS 66211

[rbharati@aafp.org](mailto:rbharati@aafp.org)

Kat Istas, MPH

American Academy of Family Physicians

11400 Tomahawk Creek Parkway

Leawood, KS 66211

[kistas@aafp.org](mailto:kistas@aafp.org)

Michael Monroe

American Academy of Family Physicians

11400 Tomahawk Creek Parkway

Leawood, KS 66211

[mmonroe@aafp.org](mailto:mmonroe@aafp.org)

**Notes about how the semi-structured interview guide was used in this study.**

The semi-structured interview guide presented here was used to help facilitate interviews with participants throughout the duration of the project. It gave the research team a central format to discuss the participants’ experiences. It was used in the group interviews at the beginning and mid-point and was used in the focus groups at the end of the project. However, the questions asked of participants varied from the interview guide based on the information emerging during the interview or based on information identified in previous interviews.

**Semi-Structure Check-In**

**INTRODUCTION:** Hello, before we begin, I would like to introduce our team and let you introduce your team. *(Introduce the AAFP team members and invite them to introduce their team members).* Before we begin, I would like to say that we are very interested in your project, regardless of where you are at. We want to hear about your successes and your challenges as there is a lot to learn from both. We also would like to record the call to help us analyze your information and pull out promising practices. Is it okay if we record this call?

**General Probes**

**(Use these throughout the interview to elicit more information)**

1. Can you tell me more about that?
2. Does anyone else have any thoughts on that?
3. **RAPPORT**: How is the project going so far?
4. **RAPPORT**: Please tell us about some of the changes that you have tried as part of the project.
5. **PLANNING**: How did you go about planning for these changes?
   - Where did you get your ideas for change from?
   - Who gave input on the ideas for change and how were they included?
   - How did you select the changes to be made?
6. **IMPLEMENTATION**: How did you implement the changes?
   - Who implemented the changes and what did they do?
   - What went well?
   - What challenges did you experience and how did you overcome them?
7. **ASSESSMENT**: How did you or will you assess if a change is successful?
   - What do you think was successful or not so successful?
   - What type of data or information did you or will you use to identify successes?
   - How will you define success in tobacco and e-cigarette prevention in a few years?
8. **INSTITUTIONALIZATION**: How will you go about institutionalizing successful changes?
   - How will you sustain successful changes?
   - How will you replicate successful changes in other parts of your practice or organization?
9. **YOUTH**: How do your processes differ between youth and adults?
10. **E-CIGARETTES**: How do your processes differ between tobacco products and electronic cigarettes?
11. **LEARNING**: What have you learned from this project?
    - Have you had any paradigm shifts or “ah-ha” moments? If yes, what were they?
12. **EXPLORATORY**: What haven’t we discussed that you think your colleagues need to know with regard to tobacco and electronic cigarette prevention and cessation?

** Semi-structured check-ins will be conducted at regular intervals throughout the project: the beginning, middle and end of the year-long implementation period. The above questions serve only as a guide for the semi-structured conversation around key themes and should not be considered comprehensive.*
